# Antigen-Specific IP-10 Release Is a Sensitive Biomarker of *Mycobacterium bovis* Infection in Cattle

**DOI:** 10.1371/journal.pone.0155440

**Published:** 2016-05-11

**Authors:** Sven D. C. Parsons, Kevina McGill, Mairead B. Doyle, Wynand J. Goosen, Paul D. van Helden, Eamonn Gormley

**Affiliations:** 1 DST/NRF Centre of Excellence for Biomedical Tuberculosis Research/SAMRC Centre for Tuberculosis Research/Division of Molecular Biology and Human Genetics, Faculty of Medicine and Health Sciences, Stellenbosch University, Cape Town, South Africa; 2 School of Veterinary Medicine, University College Dublin (UCD), Dublin, Ireland; University of Cape Town, SOUTH AFRICA

## Abstract

The most widely used ante-mortem diagnostic tests for tuberculosis in cattle are the tuberculin skin test and the interferon-gamma (IFN-γ) release assay, both of which measure cell-mediated immune responses to *Mycobacterium bovis* infection. However, limitations in the performance of these tests results in a failure to identify all infected animals. In attempting to increase the range of diagnostic tests for tuberculosis, measurement of the cytokine IP-10 in antigen-stimulated blood has previously been shown to improve the detection of *M*. *tuberculosis* and *M*. *bovis* infection, in humans and African buffaloes (*Syncerus caffer*), respectively. In the present study, 60 cattle were identified by the single intradermal comparative tuberculin test as tuberculosis reactors (n = 24) or non-reactors (n = 36) and the release of IFN-γ and IP-10 in antigen-stimulated whole blood from these animals was measured using bovine specific ELISAs. There was a strong correlation between IP-10 and IFN-γ production in these samples. Moreover, measurement of the differential release of IP-10 in response to stimulation with *M*. *bovis* purified protein derivative (PPD) and *M*. *avium* PPD distinguished between reactor and non-reactor cattle with a sensitivity of 100% (95% CI, 86%–100%) and a specificity of 97% (95% CI, 85%–100%). These results suggest that IP-10 might prove valuable as a diagnostic biomarker of *M*. *bovis* infection in cattle.

## Introduction

*Mycobacterium bovis* is the principle causative agent of bovine tuberculosis (bTB), a chronic granulomatous disease that can result in reduced productivity and death in cattle. Moreover, because *M*. *bovis*-infected animals are a potential source of infection for humans the disease is subject to comprehensive control measures in order to limit both zoonotic transmission and economic losses. Such control is typically based on test-and-slaughter schemes, which require the accurate diagnosis of infected animals.

The tuberculin skin test is the most widely used test to detect *M*. *bovis* infection in cattle; however, the more sensitive interferon-gamma (IFN-γ) release assay (IGRA) is used as an ancillary ante-mortem test [1,2]. The latter assay detects the release of IFN-γ following antigenic stimulation of whole blood with *M*. *bovis* purified protein derivative (PPDb) [1,2]. The PPDb comprises a complex mix of antigens that are not wholly specific to *M*. *bovis* and in order to take account of cross-reactive sensitization to other mycobacteria, the specificity of the assay is enhanced by comparing the PPDb-specific IFN-γ response with that in response to *M*. *avium* PPD (PPDa). Alternatively, antigens that confer superior test specificity for *M*. *bovis* and *M*. *tuberculosis*, i.e. early secretory antigenic target 6 kDa (ESAT-6) and culture filtrate protein 10 kDa (CFP-10), have been incorporated into IGRAs [3]. However, the sensitivity of these highly specific tests is lower than those using PPDb and PPDa [2,4,5].

One approach to improving the performance of such diagnostic assays is to identify additional biomarkers of immune activation. In humans, a number of alternatives to IFN-γ have been evaluated for the diagnosis of *M*. *tuberculosis* infection [6,7]. Of these, interferon gamma-induced protein 10 (IP-10) has proven particularly noteworthy [7,8]. The production of IP-10 is strongly induced by IFN-γ in human antigen presenting cells [9] and human and murine neutrophils [10,11]. Moreover, in cattle, transcription of the gene that encodes IP-10, i.e. *CXCL10*, is induced by IFN-γ in endothelial cells [12] and is highly correlated with the transcription of IFN-γ mRNA in antigen-stimulated peripheral blood mononuclear cells [13]. In bovids, ESAT-6/CFP-10 stimulation of whole blood from *M*. *bovis*-infected African buffaloes (*Syncerus caffer*) induced greater quantities of IP-10 than IFN-γ [14] and measurement of IP-10 significantly increased the sensitivity of diagnosis of this infection [15]. In contrast, in a study evaluating the diagnostic utility of this cytokine in *M*. *bovis*-infected cattle, IP-10 was not found to be a useful biomarker [13]. However, it was suggested that this might have been a consequence of limitations in the sensitivity of the enzyme-linked immunosorbent assay (ELISA) used to measure this molecule [13]. Notably, this study employed a human IP-10 ELISA [13] whereas the studies conducted in buffaloes used a commercially available bovine IP-10 assay [14,15]. Given that the measurement of antigen-induced IP-10 might increase the diagnostic sensitivity of tests of *M*. *bovis* infection, the aim of the present study was to resolve these conflicting results by re-evaluating IP-10 as a biomarker for *M*. *bovis* infection in cattle using a bovine-specific ELISA.

## Materials and Methods

### Animals

Cattle from herds naturally infected with *M*. *bovis* and cattle with no history of *M*. *bovis* exposure were tested using the single intradermal comparative tuberculin test (SICTT) as part of the national bTB eradication programme. Animals were classified as either reactors (SICTT-positive) or non-reactors (SICTT-negative) and assigned to 3 experimental groups. Group 1 consisted of twelve reactors, which were relocated and maintained at an approved research farm facility. Water and food of the highest quality were provided at all times and animals were cared for and monitored daily, with veterinary oversight according to approved standard operating procedures. There was no deviation from normal housing practices and animals were never kept in isolation. When required, cattle were humanely slaughtered at an approved slaughterhouse. Following slaughter, these animals presented with gross macroscopic lesions consistent with advanced bTB and were confirmed as bTB-positive. Hereafter, an additional 12 reactor and 16 non-reactor cattle were randomly selected from an *M*. *bovis*-infected herd in the national herd (Group 2) and were included in the study together with 20 non-reactor cattle with no known *M*. *bovis* exposure (Group 3). Heparinised blood samples for cytokine analysis were collected from all animals by a Senior Veterinary Inspector of the Irish Department of Agriculture, Food & the Marine under a research license issued by the Department of Health & Children. The study was approved by the UCD Animal Research Ethics Committee (AREC-P-11-49-Gormley) and all diagnostic testing was conducted in accordance with regulations of the EU trade Directive 64/432/EEC and the Irish bTB eradication programme.

### Interferon gamma release assay

For each animal, 1.5 ml aliquots of heparinized blood in 24-well Costar tissue culture plates (Corning Inc., Corning, NY, USA) were incubated for 24 h with, respectively, a 100 μl solution of PPDb (final conc 20 μg/ml blood) (Thermo Fisher Scientific Prionics AG, Schlieren, Switzerland), a 100 μl solution of PPDa (10 μg/ml blood) (Thermo Fisher Scientific Prionics AG), and 100 μl phosphate buffered saline (PBS) as a non-stimulating control. For all bTB-positive animals, duplicate samples were incubated as described above for 48 h and additionally, 250 μl of blood was cultured in 96-well plates (Corning Inc.) with 25 μl of PC-EC peptide cocktail at a final concentration of 5 μg/ml blood (Thermo Fisher Scientific Prionics AG) for both 24 and 48 h. All blood cultures were incubated at 37°C in a humidified atmosphere with 5% CO_2_ before harvesting of plasma supernatants following centrifugation. The production of IFN-γ in each sample was quantified in duplicate by sandwich ELISA [16] using a Bovigam^®^ ELISA kit (Thermo Fisher Scientific Prionics AG) and the relative IFN-γ concentrations were recorded as the optical density (OD) measured at 450 nm. Bovigam test results were calculated as the OD derived from the PPDb-stimulated sample minus that derived from the PPDa-stimulated sample. A Bovigam result which was greater or equal to 0.1 was regarded as positive as per the manufacturer’s recommendations.

### IP-10 ELISA

The concentration of IP-10 in plasma samples generated as described above was measured as follows. Anti-bovine CXCL10 antibody (Kingfisher Biotech Inc., St Paul, MN, USA) in PBS (1 μg/ml; 100 μl/well) was incubated overnight at 4°C in 96-well Nunc Maxisorb microtitre plates (Thermo Fisher Scientific, Waltham, MA, USA). Between subsequent steps, wells were washed with Bovigam^®^ Wash Buffer (Thermo Fisher Scientific Prionics AG) and all further reactions were done at room temperature. Wells were blocked for 1 h with 200 μl Assay Buffer consisting of 0.1% bovine serum albumin (Roche, Basel, Switzerland) and 0.05% Tween (Sigma-Aldrich, St. Louis, MO, USA) in PBS. After blocking, plasma samples (diluted 1:5 in Assay Buffer) were incubated in duplicate wells for 2 h (100 μl/well). Additionally, samples from selected cattle were incubated in parallel with a dilution series of recombinant bovine CXCL10 (Kingfisher Biotech Inc.). Hereafter, plates were incubated with biotinylated anti-bovine CXCL10 antibody (Kingfisher Biotech Inc.), diluted in Assay Buffer (0.2 μg/ml) for 1 h (100 μl/well) followed by incubation with Streptavidin-horse radish peroxidase (R & D systems, Minneapolis, MN, USA), diluted 1:200 in Assay Buffer, for 30 min (100 μl/well). After a final wash step, Bovigam^®^ Chromogen substrate was added to each well for 30 min (100 μl/well) before colour reactions were stopped with 50 μl Bovigam^®^ Stop Solution (both Thermo Fisher Scientific Prionics AG). The optical density (OD) of each reaction was measured at 450 and 620 nm. For all animals, plasma IP-10 concentrations in each sample were recorded as the OD value measured at 450 nm minus that measured at 620 nm.

### Statistical analysis

For each group of animals, all plasma samples were assayed on the same occasion which allowed for the comparison of relative IP-10 release under each experimental condition. The *M*. *bovis*-specific IP-10 release was calculated as the concentration of plasma IP-10 from whole blood stimulated with PC-EC peptides minus that in unstimulated blood (ΔEC) and the concentration of plasma IP-10 in blood incubated with PPDb corrected for the levels obtained following incubation with PPDa (ΔPPD). For selected animals, the OD values of a standard dilution series of recombinant IP-10 were used to quantify IP-10 concentrations in PPDb- and PPDa-stimulated blood samples by linear regression analysis. For these samples, the correlation between IP-10 and IFN-γ release was calculated as Spearman's rank correlation coefficient. Within animal groups, plasma IP-10 release under each experimental condition was compared using the Wilcoxon signed rank test. To investigate the diagnostic potential of IP-10, the accuracy of a test utilizing the ΔPPD results for reactor and non-reactor cattle was determined using receiver operating characteristic (ROC) curve analysis. The optimal cut off value for ΔPPD was calculated as Youden’s Index [17]. All analyses were carried out using GraphPad Prism Version 5.00 (GraphPad Software, Inc., La Jolla, CA, USA).

## Results

### Effect of antigen stimulation and incubation time on IP-10 release

**Fig 1:**
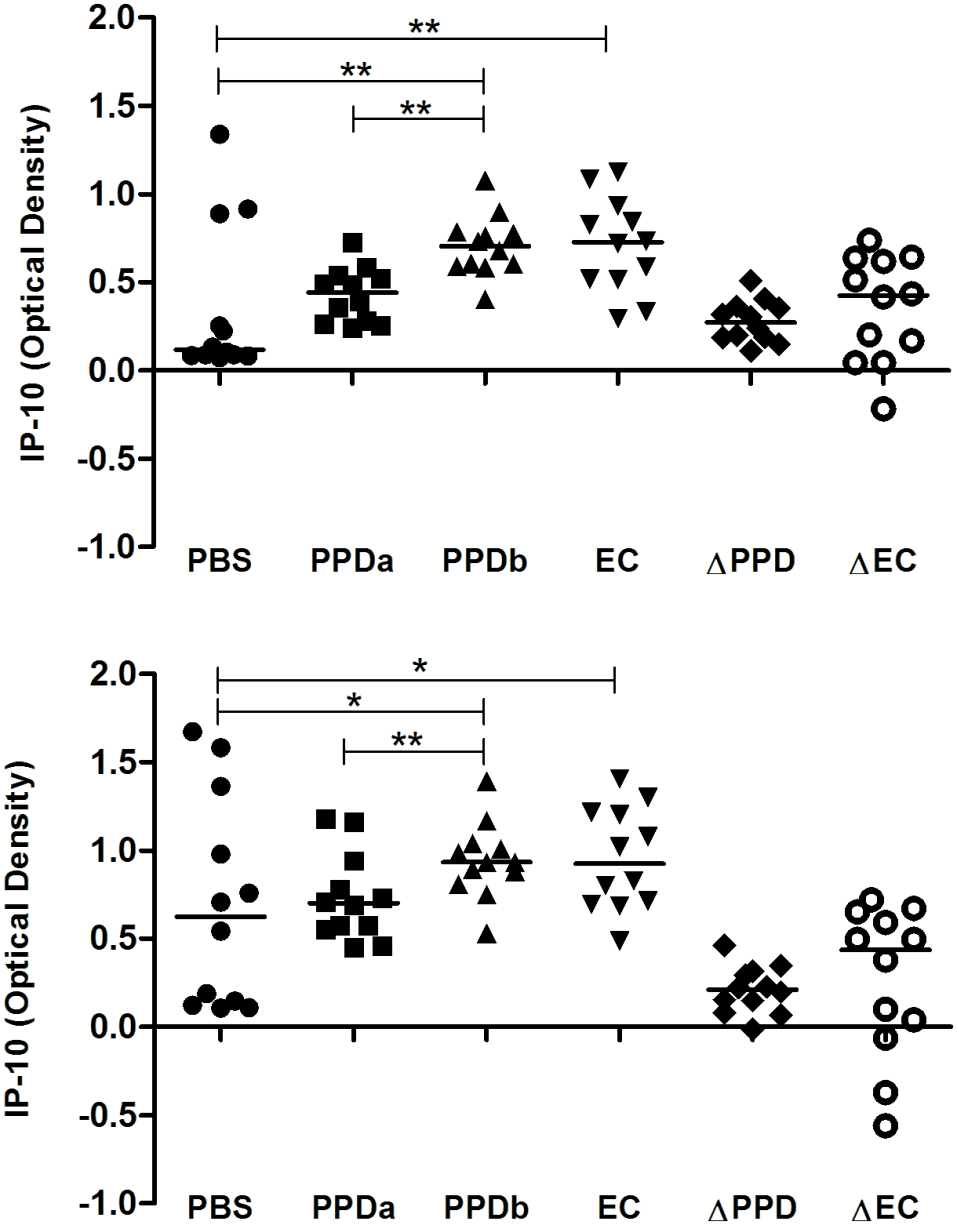
IP-10 release in response to antigenic stimulation after (A) 24 h and (B) 48 h. Whole blood from bTB-positive cattle (n = 12) was incubated at 37°C with saline (PBS), *M*. *avium* PPD (PPDa), *M*. *bovis* PPD (PPDb) and ESAT-6/CFP-10 peptides (EC). IP-10 release in whole blood in response to PPDb and EC was significantly greater than that in blood co-incubated with PBS and significantly greater in response to PPDb than in response to PPDa. Median values and the differential IP-10 responses to PPDa and PPDb (ΔPPD) and PBS and EC (ΔEC) are shown (*, p < 0.01; **, p < 0.05).

In an initial study we measured IP-10 production in whole blood from 12 bTB-positive cattle (Group 1) which was stimulated with antigen for 24 h and 48 h. Following incubation for 24 h, IP-10 release in blood stimulated with PPDb and PC-EC peptides was significantly greater when compared with that released in blood incubated with PBS (Fig 1a). The median IP-10 response in PPDb-stimulated samples was also significantly greater than in blood stimulated with PPDa. In contrast, there was no significant difference in IP-10 responses in blood samples incubated with PPDa and PBS (Fig 1a). At this time-point, all 12 bTB-positive animals showed greater IP-10 release in response to PPDb stimulation compared to PPDa stimulation while 11/12 animals showed greater IP-10 release in PC-EC peptide-stimulated blood compared with unstimulated blood (Fig 1a).

**Fig 2:**
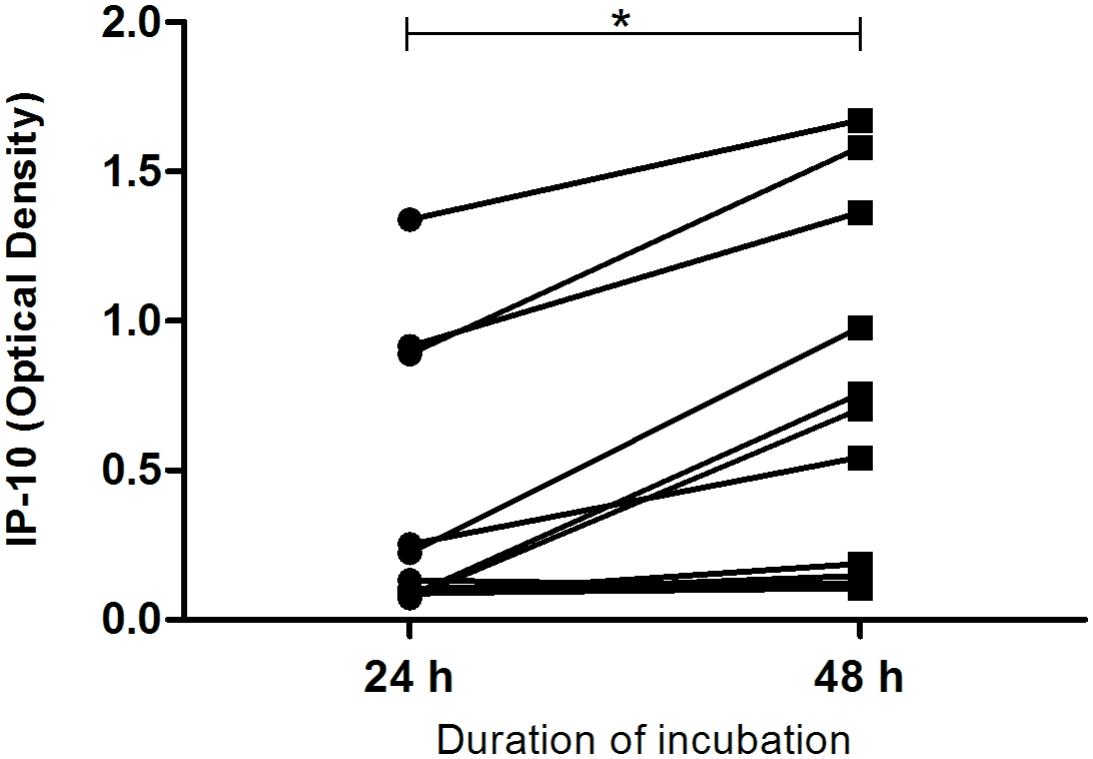
Spontaneous IP-10 release in whole blood from reactor cattle. Whole blood from cattle (n = 12) was incubated with phosphate buffered saline for 24 and 48 h. IP-10 release showed a significant increase over time (* < 0.005).

Following 48 h of incubation, median levels of IP-10 release remained significantly greater in PPDb- and PC-EC-stimulated blood compared to blood co-incubated with PBS and in blood stimulated with PPDb compared to that stimulated with PPDa (Fig 1b). At this time point, IP-10 release was greater in response to PPDb than to PPDa in 11/12 animals and greater in PC-EC peptide-stimulated blood than unstimulated blood in 9/12 animals (Fig 1b). For most animals, the release of IP-10 in blood cultured with PBS increased between 24 h and 48 h and median levels were significantly greater after 48 h of incubation when compared with the earlier time point (Fig 2).

### Antigen-specific IP-10 release in whole blood from reactor and non-reactor cattle

**Fig 3:**
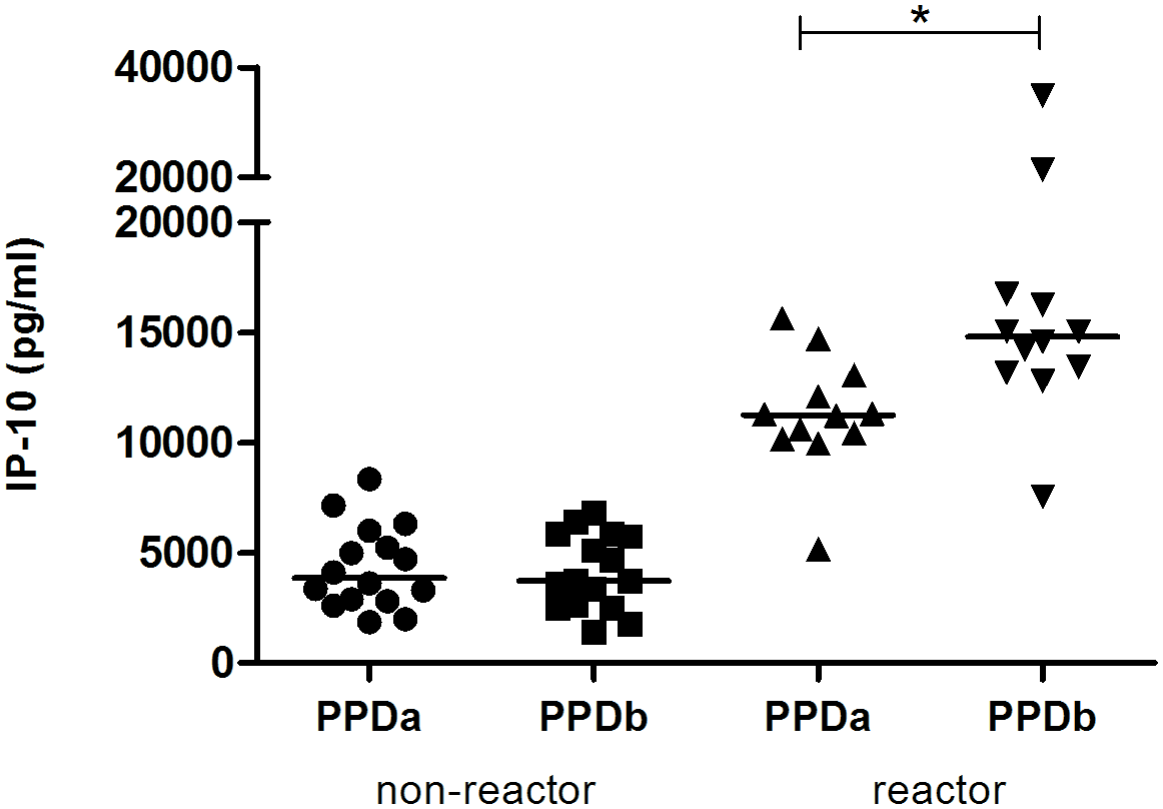
IP-10 release in response to antigenic stimulation in reactor and non-reactor cattle. Whole blood from non-reactor (n = 16) and reactor (n = 12) cattle was incubated with *M*. *avium* PPD (PPDa) and *M*. *bovis* PPD (PPDb) for 24 h at 37°C. For reactor cattle, IP-10 release in response to PPDb was significantly greater than that in response to PPDa. Median values are shown (*, p < 0.0005).

In order to further characterize the antigen-specific release of IP-10, quantitative IP-10 levels were compared in PPDb- and PPDa-stimulated blood from an additional 12 reactor and 16 non-reactor cattle from a herd with known *M*. *bovis* infection (Group 2). In the reactor group, the release of IP-10 was significantly greater in response to stimulation with PPDb (7500–34800 pg/ml) than in response to PPDa (5200–15600 pg/ml) (Fig 3). In contrast, in the non-reactor cattle, IP-10 responses to PPDb and PPDa ranged from 1400–8300 pg/ml and there was no significant difference in IP-10 responses to these antigens (Fig 3).

**Fig 4:**
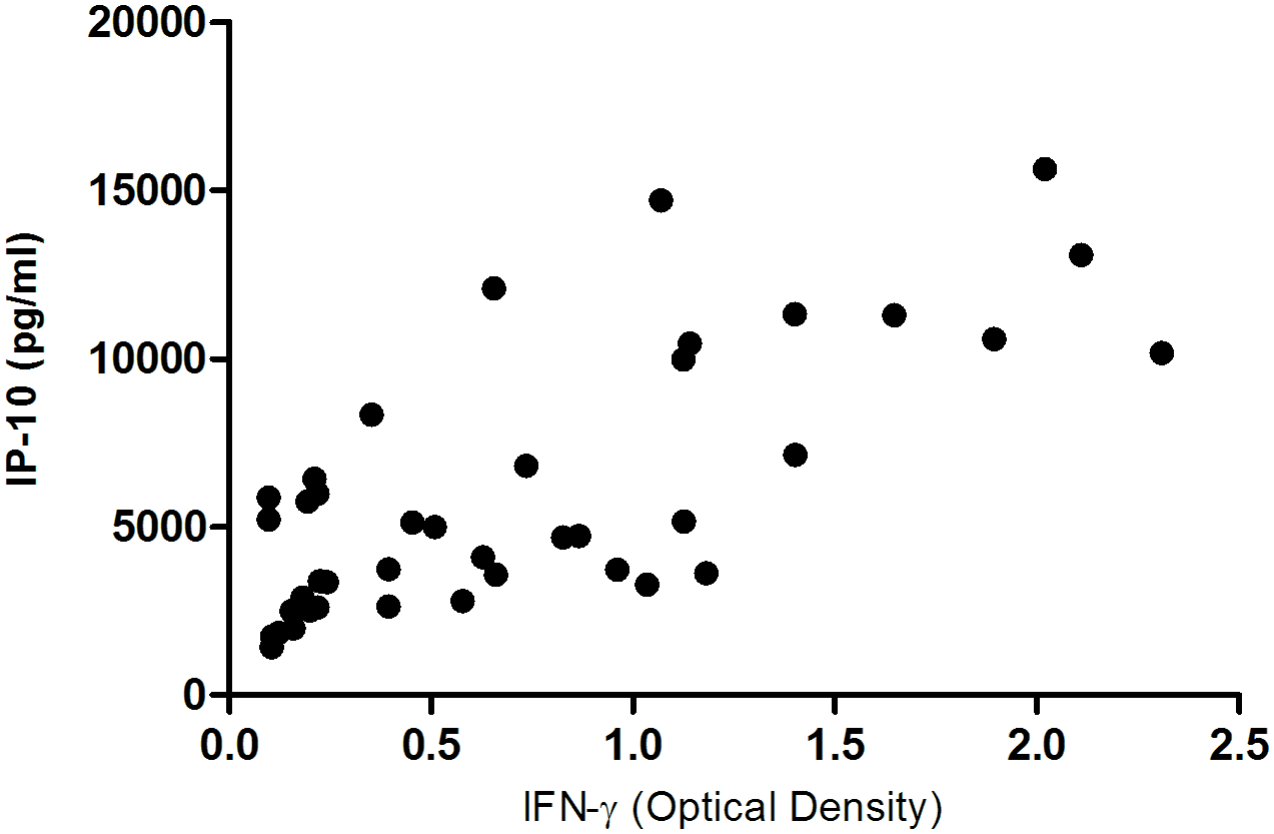
Correlation between IFN-γ and IP-10 release. Whole blood from non-reactor and reactor cattle was stimulated with either *M*. *bovis* PPD or *M*. *avium* PPD for 24 h at 37°C. The release of IFN-γ and IP-10 was highly correlated in these samples (r = 0.65; p < 0.0001).

We then investigated the relationship between IFN-γ and IP-10 release in these samples; we limited our analysis to samples with IFN-γ OD values ranging from 0.1 to 2.5 (the linear range of the ELISA). Within these limits, concentrations of IFN-γ and IP-10 were strongly correlated (r = 0.65, n = 41, p < 0.0001; Fig 4).

### Diagnostic potential of measuring *M*. *bovis*-specific IP-10 release

**Fig 5:**
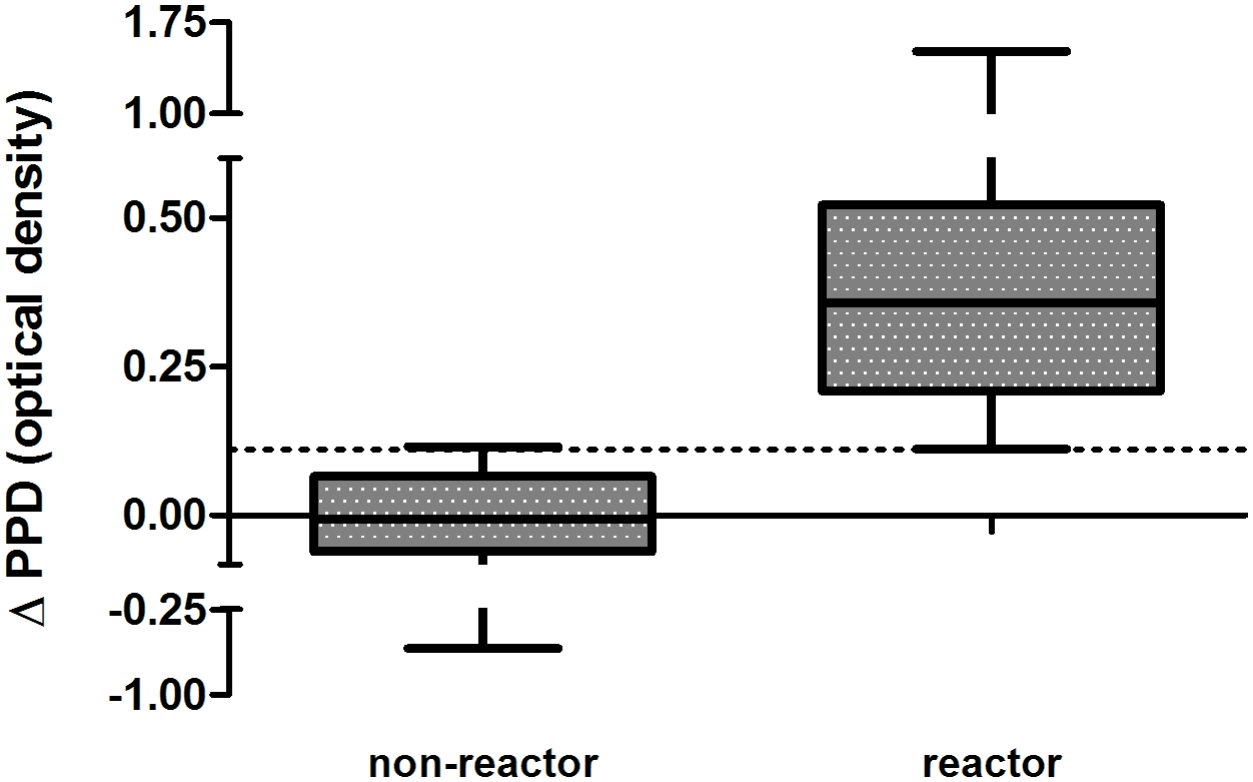
Antigen-specific IP-10 release distinguishes between non-reactor and reactor cattle. Whole blood from non-reactor (n = 36) and reactor cattle (n = 24) was incubated with *M*. *bovis* PPD and *M*. *avium* PPD for 24 h at 37°C. The difference in IP-10 release in these samples (ΔPPD) was used to calculate, by ROC analysis, an optimal cut off value of 0.11 (dotted line). This distinguished between these groups with a sensitivity of 100% and a specificity of 97%.

The potential diagnostic value of IP-10 was evaluated using ΔPPD results for all animals including an additional 20 *M*. *bovis*-unexposed cattle. All SICTT-positive cattle were Bovigam-positive and all SICTT-negative animals were Bovigam-negative. Using ROC analysis, the ΔPPD results for the 26 reactor and 36 non-reactor animals were compared and showed extremely good agreement with the Bovigam assay and SICTT status of all animals (AUC = 1.00, p < 0.0001, S1 Fig). An optimal IP-10 ΔPPD cut-off value of 0.11 distinguished between reactors and non-reactors with a sensitivity of 100% (95% CI, 86%–100%) and a specificity of 97% (95% CI, 85%–100%) (Fig 5). This cut-off value assigned all but one of the Bovigam/SICTT-negative animals as ΔPPD-negative (35/36). Changing the ΔPPD cut-off to 0.13 was the lowest value that had a specificity of 100% (95% CI, 90%– 100%) and this identified reactor cattle with a sensitivity of 96% (95% CI, 79%– 100%).

## Discussion

The most sensitive immunodiagnostic assays of *M*. *bovis* infection in animals are those that detect the activation of memory T-lymphocytes in response to stimulation with tuberculin antigens [2]. In this study we have shown that the measurement of IP-10 release in whole blood of cattle is a sensitive marker of antigen recognition and immune activation and a promising diagnostic biomarker of *M*. *bovis* infection.

The release of IP-10 was highly correlated with that of IFN-γ and after 24 h of blood culture, all reactor cattle showed consistently greater PPDb-induced IP-10 responses when compared with stimulation by PPDa. Moreover, as has been reported for humans [18] and African buffaloes [14], antigen-induced IP-10 was released at far greater concentrations than have previously been reported for IFN-γ [19,20]. Together, these findings are evidence that IP-10 is a useful measure of antigen-specific cell mediated immunity in cattle. Because IP-10 is produced in response to IFN-γ, we considered that the optimal incubation time of an assay measuring this cytokine might be different than that for an IGRA. However, in the bTB-positive cohort, the differential in the IP-10 response to PPDb and PPDa detected after 24 h of blood stimulation diminished after 48 h of incubation. It is therefore possible that a shorter incubation time might provide for even greater resolution between these responses, though this remains to be determined. Nonetheless, measurement of ΔPPD after 24 h allowed for the highly accurate discrimination between Bovigam/SICTT-positive and Bovigam/SICTT-negative animals and indicates the potential of IP-10 as a highly specific diagnostic biomarker of *M*. *bovis* infection in cattle. Notably, selection of a ΔPPD threshold with a specificity of 100%, i.e. 0.13, distinguished between these groups with a sensitivity of 96% suggesting that this marker does not significantly compromise diagnostic specificity in cattle. This finding is in agreement with human studies that have shown IP-10 assays to have a specificity of close to 100% [8]. A more sensitive cut-off value of 0.11 detected all Bovigam/SICTT-positive cattle and one additional ΔPPD-positive individual. While the true infection status of this animal was not determined, this result may indicate the potential for the IP-10 assay to increase the accuracy of diagnosis of *M*. *bovis* infection in cattle.

The release of IP-10 also shows promise as a marker of immune sensitization to the highly specific *M*. *bovis* proteins ESAT-6 and CFP-10. Importantly, measurement of IP-10, in combination with IFN-γ, has been shown to improve the detection of ESAT-6/CFP-10 sensitization in humans and African buffaloes [6,15]. In cattle, use of these antigens would be particularly useful for specifically detecting *M*. *bovis*-infected animals that have been vaccinated with *M*. *bovis* BCG [21] and for testing herds with high rates of false positive reactions to standard PPD assays [2]. However, in our study, the measurement of ESAT-6/CFP-10-specific IP-10, with reference to levels of this cytokine in unstimulated blood, was in some cases compromised by its release in control samples. The spontaneous production of IP-10 in blood from cattle has previously been reported and may reflect the *in vivo* induction of this cytokine [13]. This is consistent with findings from human studies that have identified a peripheral whole blood gene transcription signature in tuberculosis patients that is dominated by a neutrophil-driven interferon-inducible gene profile [22].

In other instances in the present study, IP-10 release in unstimulated blood exceeded that in response to ESAT-6/CFP-10 and PPD. While the mechanism of this phenomenon is unclear, the activation of antigen-specific memory lymphocytes may have resulted in reduced IP-10 production via the release of inhibitory cytokines, e.g. interleukin-10 [23]. Also, numerous proteases are secreted from activated leukocytes and proteolysis of plasma IP-10 might obscure detection of its release [24].

An earlier study with cattle experimentally infected with *M*. *bovis* found that IP-10 responses to PPDb did not exceed the respective responses to medium alone at any time point over the course of the infection [13]. This finding, in part, may have been a consequence of the high levels of IP-10 measured in unstimulated control samples in that study. However, limitations in the availability of suitable samples, and the use of a human ELISA, may have influenced these earlier findings [13]. In the present study we used a bovine-specific ELISA to measure IP-10 and showed that significant release of this protein occurs following antigenic stimulation of whole blood of cattle.

The current study was not designed to determine the diagnostic performance of the IP-10 release assay in herds undergoing episodes of tuberculosis: the animals were recruited to the study based on their skin test status. In infected herds, the IGRA is often used in parallel to the skin test in order to maximise the detection of infected animals. Whether the IP-10 test can detect additional infected animals that are undisclosed by current tests remains to be investigated. In cases where the skin test and IGRA results are inconclusive, the measurement of IP-10, which is produced in abundance in response to IFN-γ, might serve to amplify an *M*. *bovis*-specific signal in an infected animal.

In summary, IP-10 release in stimulated whole blood is strongly correlated with that of IFN-γ and is a robust biomarker of antigen-specific immunological memory in cattle. However, the spontaneous release of the cytokine in unstimulated blood requires further investigation in order to understand its origin and regulation as it might limit the performance of the assay when utilizing *M*. *bovis*-specific antigens. Nonetheless, the release of IP-10 in response to PPDb and PPDa antigens is a valuable biomarker of *M*. *bovis* infection in cattle and measurement of this cytokine might improve the diagnostic performance of whole blood stimulation assays.

## Supporting Information

S1 Fig Measurement of antigen-specific IP-10 release shows extremely good agreement with the Bovigam assay and single intradermal comparative tuberculin test (SICTT). Whole blood from Bovigam/SICTT-negative (n = 36) and Bovigam/SICTT-positive cattle (n = 24) was incubated with *M*. *bovis* PPD and *M*. *avium* PPD for 24 h at 37°C and the difference in IP-10 release in these samples (ΔPPD) was determined by ELISA. The ΔPPD results for cattle from each group were compared by ROC curve analysis and IP-10 test outcomes showed extremely good agreement with the reference tests.
(TIF)
