# Supplementary figures and images for: Antigen-Specific IP-10 Release Is a Sensitive Biomarker of Mycobacterium bovis Infection in Cattle
Source: PLoS One. 2016 May 11;11(5):e0155440. doi: 10.1371/journal.pone.0155440 (PMC4864312; doi:10.1371/journal.pone.0155440)

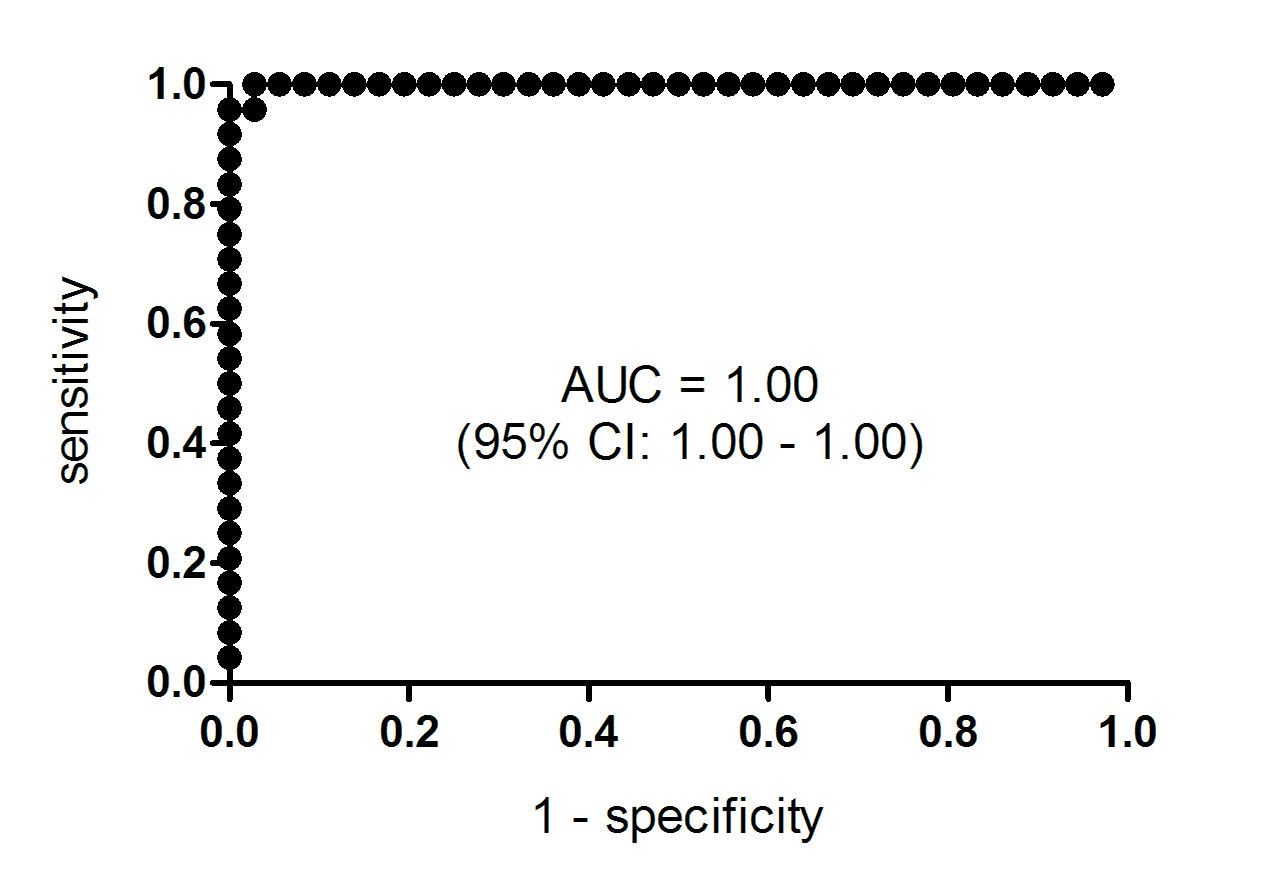

Supplement: S1 Fig — Whole blood from Bovigam/SICTT-negative (n = 36) and Bovigam/SICTT-positive cattle (n = 24) was incubated with M. bovis PPD and M. avium PPD for 24 h at 37°C and the difference in IP-10 release in these samples (ΔPPD) was determined by ELISA. The ΔPPD results for cattle from each group were compared by ROC curve analysis and IP-10 test outcomes showed extremely good agreement with the reference tests. (TIF) [file pone.0155440.s001.tif]
